# The influence of uterine leiomyomatosis on the onset of psoriasis: a nationwide population-based study of 2.5 million Korean females

**DOI:** 10.1186/s12905-024-03529-7

**Published:** 2025-02-05

**Authors:** Yeong Ho Kim, Hyun Jee Kim, Jee Yun Doh, Kyung Do Han, Ji Hyun Lee

**Affiliations:** 1 Department of Dermatology, Seoul St. Mary’s Hospital, College of Medicine, The Catholic University of Korea, Seoul, 222, Banpo-daero, Seocho-gu, Seoul, 06591 Republic of Korea; 2 Department of Dermatology, International St. Mary’s Hospital, College of Medicine, Catholic Kwandong University, 25, Simgok-ro 100 beon-gil, Seo-gu, Incheon, Republic of Korea; 3 Department of Statistics and Actuarial Science, Soongsil University, 369, Sando-ro, Dongjak- gu, Seoul, Republic of Korea

**Keywords:** Fibroid, Myomectomy, Nationwide population study, Psoriasis, Uterine leiomyomatosis

## Abstract

**Background:**

Uterine leiomyomatosis and psoriasis are prevalent conditions and shared pathophysiological factors indicate a potential association. However, a direct correlation has not been established. We investigated the relationship between uterine leiomyomatosis and the risk of new-onset psoriasis in Korean females of reproductive age.

**Methods:**

This nationwide population-based study used data from the Korean National Health Insurance System database. Data from 2,755,790 Korean females 20–39 years of age who underwent health check-ups from 2009 to 2012 were analyzed. Monitoring began at the initial national health assessment within the time frame and continued until either the diagnosis of emerging psoriasis or until December 2018.

**Results:**

Among 2,503,769 females included, 1.96% were diagnosed with psoriasis and 0.72% with uterine leiomyomatosis. The incidence ratio for new-onset psoriasis was higher in uterine leiomyomatosis patients (3.13 per 1,000) than in subjects without uterine leiomyomatosis (2.72 per 1,000). The hazard ratio for psoriasis occurrence was 1.18 (1.07–1.31) in uterine leiomyomatosis patients, 1.22 (1.08–1.37) in subjects who did not undergo myomectomy, and 1.12 (0.94–1.33) in patients who underwent myomectomy.

**Conclusions:**

Uterine leiomyomatosis patients, especially those not undergoing myomectomy, showed an increased risk of psoriasis. Lifestyle modifications and surgical intervention for uterine leiomyomatosis may also be beneficial for psoriasis occurrence.

## Introduction

Uterine leiomyomatosis (uterine fibroid or uterine myoma) is the predominant benign uterine tumor found in females of reproductive age [1]. This condition is characterized by the presence of a fibroid pseudocapsule, a complex fibro-neurovascular structure that delineates the uterine leiomyomatosis from the adjacent normal peripheral myometrium [2, 3]. Uterine leiomyomatosis can vary in size, number, and location depending on the individual [2, 4]. The incidence of uterine leiomyomatosis widely varies across studies and geographical regions (4.5–68.6%) and this variation can be attributed to differences in the nature of the investigation, the diagnostic techniques used, and the racial or ethnic composition of the populations studied [5, 6]. Although a benign tumor, uterine leiomyomatosis can become an indication for hysterectomy in some cases, and can cause menorrhagia, pelvic pain, infertility, recurrent miscarriage, and preterm labor [2, 7, 8]. Although uterine leiomyomatosis is typically benign, misdiagnosis of uterine leiomyosarcoma, a rare but aggressive malignancy, can occur due to overlapping symptoms such as abnormal bleeding and pelvic masses [9]​. Accurate differentiation is critical as leiomyosarcomas require more invasive treatments like hysterectomy, while leiomyomas may be managed conservatively [9]. Raffone et al. (2023) reported that ultrasound alone has moderate diagnostic accuracy, with 76% sensitivity and 89% specificity, emphasizing the need for a more comprehensive diagnostic approach, including imaging and biopsy, to avoid inappropriate treatments [9]​.

The pathophysiology of uterine leiomyomatosis involves the transformation of smooth muscle stem cells (myometrial stem cells) within the myometrium [7, 10]. This transformation occurs through the paracrine mechanisms mediated by estrogen, progesterone, and WNT/β-catenin pathway, forming fibroid progenitor cells [7, 11]. In addition, various growth factors such as basic fibroblast growth factor (bFGF), vascular endothelial growth factor (VEGF), and insulin-like growth factor (IGF) [12], exert effects on the myometrium and leiomyoma, contributing to the pathophysiology of uterine leiomyomatosis. Risk factors for uterine leiomyomatosis include older age, race, nulliparity, premenopause, family history of uterine leiomyomatosis, hypertension (HTN), food additives, soy milk, obesity, low vitamin D levels, high vitamin E levels, microbiome change, endocrine-disrupting chemicals, smoking, and alcohol consumption [2, 13–25].

Psoriasis is a chronic inflammatory skin disease that appears primarily as well-demarcated, erythematous scaly plaques predominantly manifesting in symmetrical patterns on elbows, knees, trunk, and scalp [26–28]. Globally, the prevalence rate is approximately 2%, with reported cases of over 60 million adults and children affected [29–32]. Psoriasis is triggered by the activation of plasmacytoid dendritic cells due to genetic and environmental factors, leading to the production of various proinflammatory cytokines, including tumor necrosis factor (TNF)-α, interferon (IFN)-γ, interleukin (IL)-17, IL-22, IL-23, and IL-1β [33], resulting in chronic inflammation through excessive keratinocyte proliferation [26, 34]. The risk factors for psoriasis include mechanical stress, smoking, alcohol consumption, air pollution, metabolic syndrome, obesity, HTN, diabetes mellitus (DM), dyslipidemia, drugs, vaccination, infection, and mental stress [27, 29].

Although direct correlations between uterine leiomyomatosis and psoriasis have not been reported to date, emerging evidence indicates a potential association [35–37]. Therefore, in the present study, the association between uterine leiomyomatosis and the likelihood of developing new-onset psoriasis among Korean females of reproductive age was investigated utilizing a comprehensive national health screening cohort.

## Methods

### Study design and database

This retrospective cohort study utilized the Korean National Health Insurance System (K-NHIS) to analyze data from a population-based cohort. Data were collected from the K-NHIS that contains invoicing records of healthcare professionals and includes details on patient age, gender, demographic features, past treatments, overall health evaluations, lifestyle, and habits. Medical professionals are recommended to perform regular medical checks either every 2 years or annually. Korea has more than 50 million residents and subscribing to the K-NHIS is mandatory for all citizens [38–40].

### Study population

**Fig. 1:**
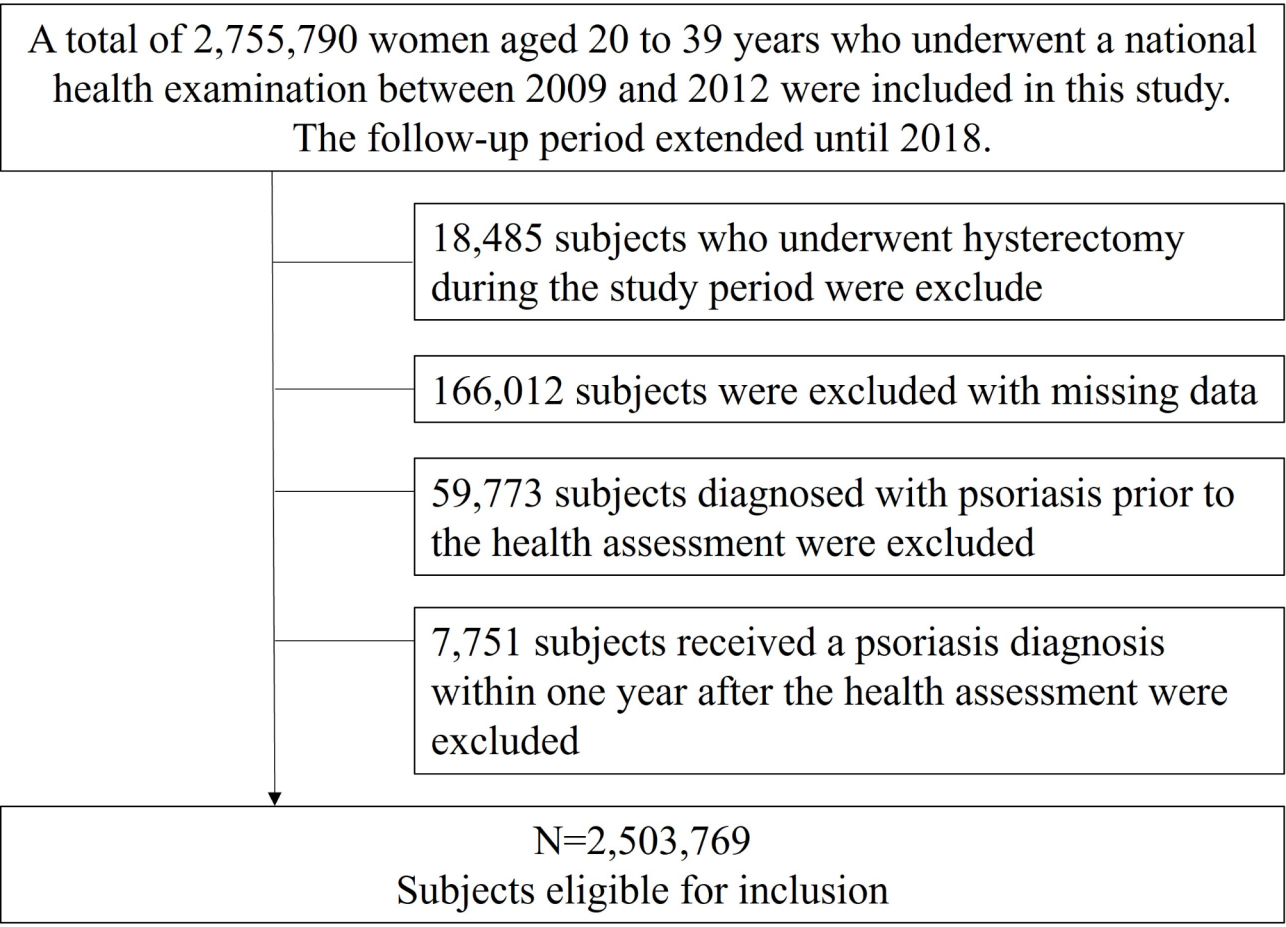
Study flow chart

Data from 2,755,790 Korean females 20–39 years of age who underwent health check-ups from 2009 to 2012 were analyzed. The 20–39 age range was selected as it represents the primary reproductive age group for women and has a high incidence of uterine leiomyomatosis. Inclusion criteria involved females diagnosed with uterine leiomyomatosis within the designated period, with no prior history of psoriasis. Uterine leiomyomatosis was defined based on the International Classification of Diseases, Tenth Revision (ICD-10) code D25 in the K-NHIS database [41], while psoriasis was defined by the ICD-10 code L40 [42]. A diagnosis of psoriasis was confirmed by having either two outpatient records or a single inpatient record with these codes within a year. Participants who underwent hysterectomy during the analysis period, were previously diagnosed with psoriasis during screening period (from January 2002 to their initial health check-up), were diagnosed with psoriasis within 1 year of the starting examination to account for a latency period, or had incomplete data on uterine leiomyomatosis were excluded. Monitoring for the development of psoriasis began at the initial national health assessment within the time frame and continued until either the diagnosis of emerging psoriasis or until December 2018. Consequently, the present study included 2,503,769 females 20–39 years of age. The flowchart depicting the inclusion process for the study population is shown in Fig. 1. Due to the retrospective nature of the study and the reliance on anonymous databases and publicly available medical records, the Institutional Review Board at Seoul St. Mary’s Hospital, Catholic University of Korea approved the study (KC22ZISI0317).

### Measurements and definitions

Body mass index (BMI) was determined by dividing patient weight in kilograms by the square of height in meters (kg/m^2^). A BMI ≥ 25 kg/m^2^ was defined as obesity [43, 44] . Household income was divided into two categories: income in the bottom quartile based on monthly contributions to the National Health Insurance Corporation and the remainder [44, 45].

The initial comorbid conditions of the study population were identified using the ICD-10 classification codes. The following ICD-10 codes were used: uterine leiomyomatosis (D25), DM (E10–E14), HTN (I10–I15), dyslipidemia (E78), stroke (I63, I64), and ischemic heart ailments (I20–I25). For DM, the criteria were either a minimum of one yearly claim under ICD-10 codes E10–E14 and one yearly claim for antidiabetic medicine prescription, or a fasting blood sugar level ≥ 126 mg/dL. HTN was defined as either a minimum of one yearly claim under ICD-10 codes I10–I15 and one yearly claim for antihypertensive drug prescription, or a blood pressure measurement ≥ 140/90 mm Hg. Dyslipidemia was defined as either at least one yearly claim under ICD-10 code E78 and one yearly claim for cholesterol-lowering drug prescription, or an overall cholesterol measure ≥ 240 mg/dL. Any other health complications were noted if one or multiple diagnostic entries were available.

Social behaviors including smoking habits, alcohol intake, and physical exertion, were determined using personal disclosure questionnaires. For smoking habits, classification included current smoker, ex-smoker, or never-smoker based on responses. A current smoker was considered a person currently smoking and who had consumed > 5 packs or a cumulative of 100 cigarettes in their lifetime. An ex-smoker was considered a subject who previously consumed > 5 packs but had since quit by the time of the questionnaire. Never-smokers were individuals who had consumed ≤ 5 packs [44, 46]. Regarding alcohol intake, individuals consuming ≥ 30 g daily were considered heavy drinkers and subjects consuming < 30 g daily were mild to moderate drinkers. Individuals participating in intense physical exercise for over 20 min at least three times a week, or moderate exercise for over 30 min at a minimum of five times a week, were considered to participate in regular physical activity.

### Study outcomes and follow-up

In the present study, a psoriasis diagnosis was confirmed by having either two outpatient records or a single inpatient record under the ICD-10 code for psoriasis (L40) in the K-NHIS database within 1 year following the index examination. The primary endpoint of the investigation was to identify the emergence of new-onset psoriasis cases. The incidence rate of new-onset psoriasis was determined as the count of new cases per 1,000 patient-years of follow-up. Furthermore, how uterine leiomyomatosis influences the onset of psoriasis was investigated.

### Statistical analysis

Numerical data are represented as the mean ± standard deviation (SD) and/or as the geometric mean within a 95% confidence interval (CI). Countable data are depicted through counts and their corresponding percentages. The Student’s *t*-test was used to assess differences in continuous variables and the chi-square test for categorical variables. We used Cox proportional hazards models to calculate hazard ratios (HR) for the development of psoriasis, which accounts for the time-to-event nature of the data and allows for varying follow-up times among participants, estimating the HR and its 95% CI for each independent factor. Model 1 remained unadjusted and the multifactorial regression Model 2 factored in age, smoking status, alcohol consumption, and consistent physical activity. Model 3 incorporated all components of Model 2 along with DM, HTN, dyslipidemia, chronic kidney disease (CKD), and BMI. The Kaplan-Meier method was used for the HRs for uterine leiomyomatosis, considering both surgical and no surgical interventions. All statistical calculations were conducted using the SAS software (version 9.4; SAS Institute Inc., Cary, NC, USA) with *P*-values ≤ 0.05 indicative of statistical significance.

## Results

### Baseline characteristics of the study population

Among the 2,503,769 females included in the analysis, 49,177 (1.96%) were diagnosed with psoriasis during the study period, 18,025 (0.72%) were diagnosed with uterine leiomyomatosis, and 5,854 (0.23%) underwent myomectomy.

The baseline characteristics of the study population are summarized in Table 1. Among individuals 20–29 years of age, 26,511 (2.04%) were diagnosed with psoriasis compared with 22,666 (1.89%) subjects 30–39 years of age. Furthermore, a higher incidence of psoriasis was observed in cases with a medical history of coexisting conditions such as uterine leiomyomatosis (406 cases, 2.25%), DM (557 cases, 2.37%), HTN (1,238 cases, 2.11%), dyslipidemia (1,999 cases, 2.15%), and CKD (1,571 cases, 2.28%). The incidence of psoriasis was increased in individuals who smoked (3,490 cases, 2.35% in current smokers) and consumed alcohol (1,355 cases, 2.27% in heavy drinkers). Conversely, subjects in the lowest 25% income bracket exhibited a lower incidence of psoriasis (10,393 cases, 1.91%).

**Table 1:** Baseline characteristics of subjects

		Psoriasis		
	Total participants	No	Yes	*P*-value
N	2,503,769	2,454,592	49,177	
Age (years) ± SD	29.71 ± 5.22	29.72 ± 5.22	29.48 ± 5.13	< 0.0001
20–29 (%)	1,301,692 (51.99)	1,275,181 (51.95)	26,511 (53.91)	< 0.0001
30–39 (%)	1,202,077 (48.01)	1,179,411 (48.05)	22,666 (46.09)	
Leiomyoma				0.0051
No (%)	2,485,744 (99.28)	2,436,973 (99.28)	48,771 (99.17)	
Yes (%)	18,025 (0.72)	17,619 (0.72)	406 (0.83)	
Myomectomy				0.3447
No (%)	2,497,915 (99.77)	2,448,863 (99.77)	49,052 (99.75)	
Yes (%)	5,854 (0.23)	5,729 (0.23)	125 (0.25)	
DM (%)	23,497 (0.94)	22,940 (0.93)	557 (1.13)	< 0.0001
HTN (%)	58,621 (2.34)	57,383 (2.34)	1,238 (2.52)	0.0091
Dyslipidemia (%)	93,060 (3.72)	91,061 (3.71)	1,999 (4.06)	< 0.0001
Smoking status				< 0.0001
Never-smoker (%)	2,265,750 (90.49)	2,221,940 (90.52)	43,810 (89.09)	
Ex-smoker (%)	89,684 (3.58)	87,807 (3.58)	1,877 (3.82)	
Current smoker (%)	148,335 (5.92)	144,845 (5.9)	3,490 (7.1)	
Alcohol consumption				< 0.0001
Non	1,365,076 (54.52)	1,338,572 (54.53)	26,504 (53.9)	
Mild to moderate	1,078,934 (43.09)	1,057,616 (43.09)	21,318 (43.35)	
Heavy	59,759 (2.39)	58,404 (2.38)	1,355 (2.76)	
BMI (kg/m^2^) ± SD	21.35 ± 3.25	21.35 ± 3.25	21.45 ± 3.35	< 0.0001
< 18.5 (%)	371,744 (14.85)	364,722 (14.86)	7,022 (14.28)	< 0.0001
< 23 (%)	1,545,831 (61.74)	1,515,534 (61.74)	30,297 (61.61)	
< 25 (%)	290,864 (11.62)	285,152 (11.62)	5,712 (11.62)	
< 30 (%)	236,637 (9.45)	231,775 (9.44)	4,862 (9.89)	
≥ 30 (%)	58,693 (2.34)	57,409 (2.34)	1,284 (2.61)	
Regular physical activity (yes) (%)	239,015 (9.55)	234,206 (9.54)	4,809 (9.78)	0.0761
Low income (25%) (%)	545,332 (21.78)	534,939 (21.79)	10,393 (21.13)	0.0005
CKD (GFR < 60) (%)	68,791 (2.75)	67,220 (2.74)	1,571 (3.19)	< 0.0001
GFR (mL/min/1.73 m^2^) ± SD	98.3 ± 41.29	98.3 ± 41.31	98.06 ± 40.04	0.2019
Glucose (mg/dL) ± SD	88.17 ± 13.19	88.17 ± 13.17	88.23 ± 13.94	0.3754
SBP (mm Hg) ± SD	111.24 ± 11.53	111.24 ± 11.53	111.26 ± 11.53	0.645
DBP (mm Hg) ± SD	69.85 ± 8.53	69.84 ± 8.53	69.86 ± 8.55	0.749
HDL-C (mg/dL) ± SD	63.22 ± 23.56	63.23 ± 23.54	63.12 ± 24.11	0.3244
LDL-C (mg/dL) ± SD	99.22 ± 30.91	99.22 ± 30.91	99.05 ± 31.29	0.2046

Acronyms: BMI, body mass index; CKD, chronic kidney disease; DBP, diastolic blood pressure; DM, diabetes mellitus; GFR, glomerular filtration rate; HDL-C, high-density lipoprotein cholesterol; HTN, hypertension; LDL-C, low-density lipoprotein cholesterol; SBP, systolic blood pressure; SD, standard deviation

### Incidence and association of psoriasis with uterine leiomyomatosis and myomectomy status

In subjects without uterine leiomyomatosis, the incidence ratio for new-onset psoriasis was 2.72 per 1,000 individuals (48,771 cases out of 2,485,744 individuals), and among uterine leiomyomatosis patients, this ratio was higher at 3.13 per 1,000 individuals (406 cases out of 18,025 individuals). In multivariate analysis, after adjusting for age, smoking status, alcohol consumption, regular physical activity, DM, HTN, dyslipidemia, CKD, and BMI, the HR for psoriasis occurrence was 1.18 (95% CI: 1.07–1.31) in uterine leiomyomatosis patients (Table 2, Model 3). The HR was 1.22 (95% CI: 1.08–1.37) in the uterine leiomyomatosis patients who did not undergo myomectomy (281 cases out of 12,717 individuals), and 1.12 (95% CI: 0.94–1.33) in the uterine leiomyomatosis patients who did undergo myomectomy (125 cases out of 5,854 individuals) (Table 3, Model 3).

**Table 2:** Risk of new-onset psoriasis associated with uterine leiomyomatosis

Uterine leiomyomatosis	*N*	Psoriasis	IR per 1,000	Model 1	*P*-value	Model 2	*P*-value	Model 3	*P*-value
No	2,485,744	48,771	2.72	1 (Ref.)	0.0053	1 (Ref.)	0.0007	1 (Ref.)	0.0007
Yes	18,025	406	3.13	1.15 (1.04–1.27)		1.18 (1.07–1.31)		1.18 (1.07–1.31)	

Acronyms: IR, incidence rate; DM, diabetes mellitus; HTN, hypertension; CKD, chronic kidney disease; BMI, body mass index

Model 1: not adjusted

Model 2: adjusted for age, smoking status, alcohol consumption, and regular physical activity

Model 3: adjusted for age, smoking status, alcohol consumption, regular physical activity, DM, HTN, dyslipidemia, CKD, and BMI

**Table 3:** Risk of new-onset psoriasis based on uterine leiomyomatosis and myomectomy status

Uterine leiomyomatosis, myomectomy status	*N*	Psoriasis	IR per 1000	Model 1	*P*-value	Model 2	*P*-value	Model 3	*P*-value
No	2,485,744	48,771	2.72	1 (Ref.)	0.0144	1 (Ref.)	0.0023	1 (Ref.)	0.0023
Yes, without myomectomy	12,717	281	3.21	1.18 (1.05–1.33)		1.22 (1.08–1.37)		1.22 (1.08–1.37)	
Yes, with myomectomy	5,854	125	2.95	1.09 (0.91–1.29)		1.12 (0.94–1.33)		1.12 (0.94–1.33)	

Acronyms: IR, incidence rate; DM, diabetes mellitus; HTN, hypertension; CKD, chronic kidney disease; BMI, body mass index

Model 1: not adjusted

Model 2: adjusted for age, smoking status, alcohol consumption, and regular physical activity

Model 3: adjusted for age, smoking status, alcohol consumption, regular physical activity, DM, HTN, dyslipidemia, CKD, and BMI

**Fig. 2:**
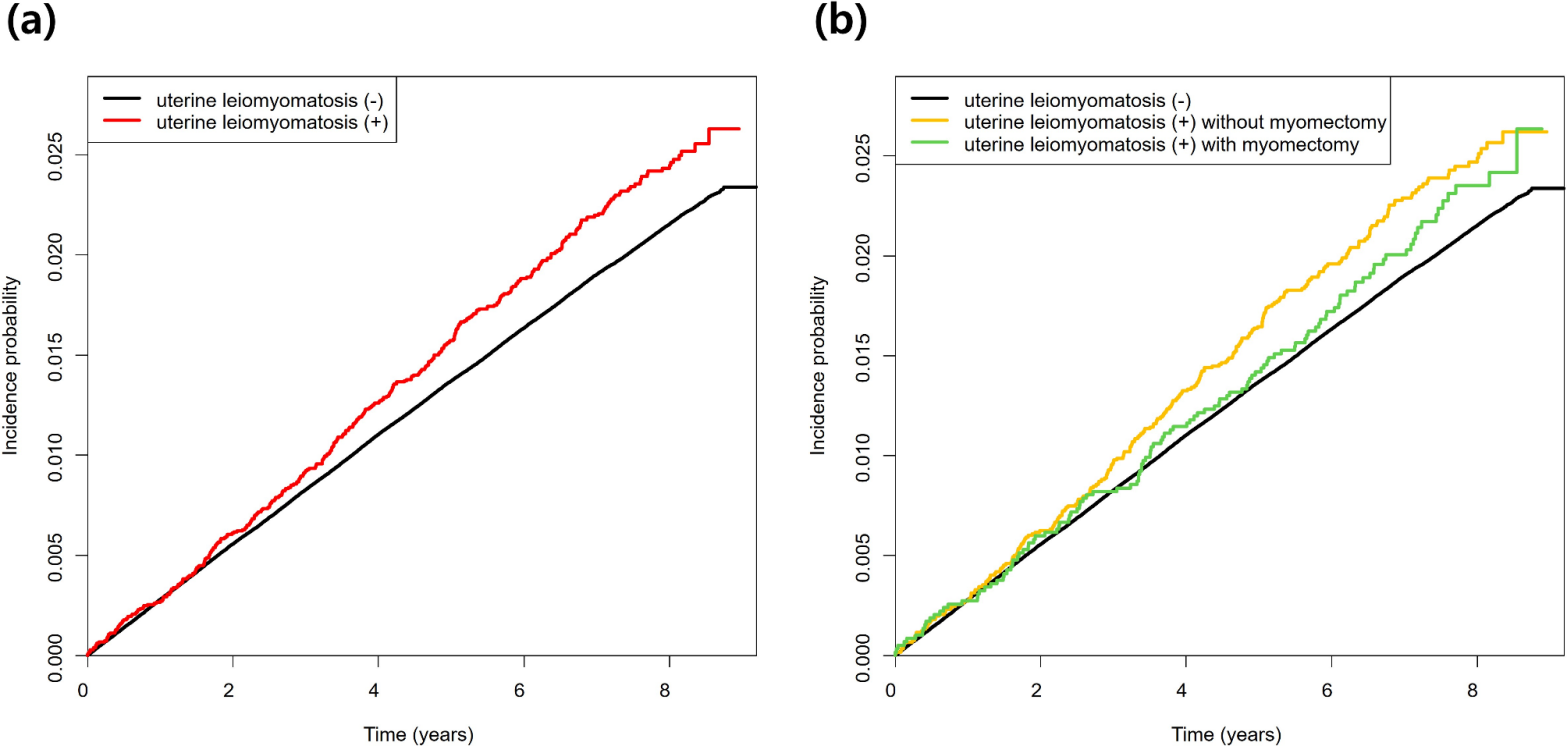
Kaplan-Meier curves of psoriasis incidence by uterine leiomyomatosis (**a**) and myomectomy status (**b**)

In the Kaplan-Meier survival assessment where the outcome was a diagnosis of psoriasis, the uterine leiomyomatosis patients who did not undergo myomectomy had the lowest rate of disease-free survival, with a cumulative incidence of psoriasis of approximately 3.5% at 10 years follow-up, compared to approximately 3% for those who underwent myomectomy and 2.5% for those without uterine leiomyomatosis (Fig. 2).

## Discussion

In the present study, results showed an increased risk of psoriasis in patients with uterine leiomyomatosis, and a more increased risk of psoriasis in patients with uterine leiomyomatosis who did not undergo myomectomy. A potential association between these conditions exists due to shared risk factors such as HTN, obesity, smoking, and alcohol consumption.

Studies examining the association between uterine leiomyomatosis and immune system-related diseases are relatively scarce [35]. The relationship with systemic lupus erythematosus (SLE) remains unclear, with one small study showing a lower SLE prevalence in women with uterine fibroids compared to those with endometriosis [36]. However, women with endometriosis have an increased risk of developing uterine fibroids [37], suggesting a complex interrelationship. Research on thyroid disorders shows mixed results, with some studies indicating an increased risk of autoimmune thyroid disorders in women with endometriosis [35, 47]. Similarly, the association with rheumatoid arthritis (RA) is inconsistent, with some studies reporting an increased risk [48] and others finding no significant association [49]. A large case-control study found a significantly increased risk of inflammatory bowel disease (IBD) in pregnant women with a history of endometriosis (OR = 2.06, 95% CI: 1.83–2.31) [50]. Similarly, a cohort study reported an increased incidence of IBD in women with endometriosis (SIR = 1.5, 95% CI: 1.4–1.7) [51]. However, these studies primarily focused on endometriosis rather than uterine leiomyomatosis specifically [35]. Overall, the quality of evidence for these associations is generally low, and further large-scale, well-designed studies are needed to clarify the relationship between uterine leiomyomatosis and immune system-related diseases [35].

Furthermore, the pathophysiology of uterine leiomyomatosis and psoriasis could be associated through common pathways such as WNT/β-catenin, TNF-α, VEGF, and bFGF.

In the pathophysiology of uterine leiomyomatosis, the transformation of smooth muscle stem cells occurs through paracrine mechanisms mediated by the WNT/β-catenin pathway. The increased growth observed in fibroids with mutations in the mediator complex subunit 12 (MED12) may be explained by the interaction between estrogen and the WNT/β-catenin pathway, combined with TGF-β [7]. Furthermore, mutations in MED12 have been identified in rare fibroid variations, including atypical, cellular, and lipoleiomyomas in addition to leiomyosarcomas and smooth muscle tumors with uncertain malignant potential [7, 52]. TNF-α is a cytokine secreted from activated macrophages and plays a role in regulating inflammation, immunity, cell growth and differentiation, and apoptosis. The TNF-α protein expression was higher in uterine leiomyomatosis compared with normal myometrial tissue [53–55]. In addition, a more pronounced VEGF expression was observed in leiomyomas compared with the adjacent myometrium indicating that local angiogenesis might play a pivotal role in the tumorigenesis and growth of these tumors [56]. Furthermore, the expression of bFGF and its receptors, fibroblast growth factor receptor (FGFR)-1 and FGFR-2, was found in both leiomyomas and myometrial cells in previous studies [12, 57–62]. Notably, a more pronounced FGFR-1 expression was observed in the tumors compared with the myometrium [62].

Evidence has been provided in multiple investigations for the involvement of the WNT/β-catenin pathway and TNF-α in the pathophysiology of psoriasis. In a previous study, psoriasis lesions were investigated through biopsy and examining WNT molecules of the WNT/β -catenin pathway and the authors determined that Wnt5a levels were elevated 4-fold in the lesional skin [63]. In another study in which Wnt5a was investigated, knockdown of Wnt5a in cultured HaCaT keratinocytes and normal human keratinocytes suppressed keratinocyte proliferation and induced apoptosis [64]. TNF-α is notably upregulated in the keratinocytes present in psoriatic lesions [65]. TNF-α, produced by mast cells, macrophages, keratinocytes, and lymphocytes, apparently increases the expression of IL-8, VEGF, bFGF, angiopoietin, and Tie-2 (angiopoietin receptor) receptor in endothelial cells [66, 67]. Patients with psoriasis exhibit elevated serum VEGF levels, and a notable correlation exists between the severity of psoriasis and these VEGF levels [68–70]. Furthermore, the increased VEGF expression in skin samples from individuals with psoriasis [71] emphasizes its pivotal role in preserving the integrity of the epidermal barrier [72]. This connection between epidermal VEGF and keratinocyte proliferation indicates a significant involvement of VEGF in the proliferation of keratinocytes [72–74].

Notably, among individuals with uterine leiomyomatosis, the patients that underwent myomectomy did not exhibit a significant increase in the HR for the occurrence of psoriasis. In contrast, the patients that did not undergo myomectomy showed a statistically significant increase in HR for psoriasis development. These results are due to the changes in cytokines that occur after myomectomy. In a recent study, TNF-α levels decreased in patients with uterine intramural leiomyoma following myomectomy as evidenced by endometrial sampling [75]. In another study in which the treatment of uterine leiomyomatosis was investigated using mifepristone and ultrasound-guided radiofrequency ablation, the authors observed a significant reduction in the serum levels of VEGF, epidermal growth factor (EGF), bFGF, TGF-β, and TGF-β receptor among patients after either ultrasound-guided radiofrequency ablation or the combined approach of oral administration of mifepristone with ultrasound-guided radiofrequency ablation [76]. Changes in these cytokines can occur even after myomectomy, and consequently, may influence the onset of psoriasis.

The present study had several limitations. In the process of including the research population, individuals who underwent hysterectomy during the study period were excluded. However, in the situation of uterine leiomyomatosis patients experiencing heavy menstrual bleeding and no longer desiring pregnancy, hysterectomy might have been performed [7]. Consequently, a potential exists for the exclusion of such patients from the study. Furthermore, because the data were based on medical records of patient hospital visits, a bias arising from the absence of records may exist for individuals with diseases such as uterine leiomyomatosis or psoriasis if they did not seek medical treatment. Our study is limited by the lack of histological confirmation in defining uterine leiomyomatosis, relying instead on ICD-10 codes from medical records. Additionally, the specific diagnostic methods used (e.g., ultrasound, magnetic resonance imaging) are not available in this database, which further limits the precision of the diagnosis. This approach, while enabling a large-scale population study, may have led to some misclassification of cases. We were unable to adjust for potential confounding factors such as medication use (e.g., propranolol) or certain medical conditions (e.g., hyperthyroidism) that may influence psoriasis development. Future studies should aim to include these variables in their analyses. Another limitation of this study is that we did not account for the potential delayed diagnosis of uterine leiomyomatosis. Our study design, which used a fixed baseline period for leiomyomatosis diagnosis, may have missed cases diagnosed later in the follow-up period. Future studies should consider time-varying exposure analyses to address this potential bias and capture diagnoses of leiomyomatosis that occur throughout the study period. Additionally, future studies should extend this analysis to women aged 40–59 and consider stratification by menopausal status to provide a more comprehensive understanding of the association between uterine leiomyomatosis and psoriasis across different age groups and hormonal states. However, considering the age of onset of psoriasis, it was thought that focusing on subsequent psoriasis in individuals with leiomyomatosis aged 20–39 could yield more worthy results.

Despite the limitations, the present study had several strengths. The results provide significant contributions. A comprehensive national database representative of all Korean women of reproductive age was used to explore the relationship between uterine leiomyomatosis and psoriasis. To the best of our knowledge, this is the first research in which the association between uterine leiomyomatosis and the likelihood of developing psoriasis was investigated. Furthermore, by comparing the HR of psoriasis based on the myomectomy status, the importance of surgical treatment for uterine leiomyomatosis was substantiated. From a clinical perspective, practitioners should be aware of the potential association between these conditions. When treating patients with psoriasis, especially women of reproductive age, clinicians might consider screening for uterine leiomyomatosis. Conversely, women diagnosed with leiomyomatosis should be informed about the slightly increased risk of developing psoriasis and advised to report any skin changes promptly.

## Conclusions

In conclusion, among female patients with uterine leiomyomatosis, the risk of developing psoriasis was higher compared with subjects without uterine leiomyomatosis. Furthermore, the risk of psoriasis was greater in leiomyoma patients without myomectomy. Therefore, adopting lifestyle modifications (e.g., quitting smoking and drinking alcohol) and preventing accompanying conditions such as HTN, diabetes, and hyperlipidemia, is important. In addition, performing myomectomy for uterine leiomyomatosis may be beneficial for psoriasis occurrence; however, additional studies are needed to elucidate the underlying processes.

## Data Availability

Availability of data and materialsThe datasets analysed during the current study are available from the NHIS data sharing service data (https://nhiss.nhis.or.kr/bd/ab/bdaba000eng.do).

## Abbreviations

BMI: Body mass index
bFGF: Basic fibroblast growth factor
CI: Confidence interval
CKD: Chronic kidney disease
DM: Diabetes mellitus
EGF: Epidermal growth factor
FGFR: Fibroblast growth factor receptor
HR: Hazard ratio
HTN: Hypertension
ICD: 10-International classification of diseases, tenth revision
IFN: Interferon
IGF: Insulin-like growth factor
IL: Interleukin
K: NHIS-Korean national health insurance system
MED12: Mediator complex subunit 12
SD: Standard deviation
TGF: β-Transforming growth factor beta
TNF: Tumor necrosis factor
VEGF: Vascular endothelial growth factor
